# The challenge of developmental therapeutics for adrenocortical carcinoma

**DOI:** 10.18632/oncotarget.8774

**Published:** 2016-04-18

**Authors:** Ricardo Costa, Benedito A. Carneiro, Fabio Tavora, Sachin G. Pai, Jason B. Kaplan, Young Kwang Chae, Sunandana Chandra, Peter A. Kopp, Francis J. Giles

**Affiliations:** ^1^ Northwestern Medicine Developmental Therapeutics Institute, Robert H. Lurie Comprehensive Cancer Center of Northwestern University, Chicago, IL, USA; ^2^ Division of Hematology and Oncology, Feinberg School of Medicine, Northwestern University, Chicago, IL, USA; ^3^ Division of Endocrinology, Metabolism, and Molecular Medicine, Feinberg School of Medicine, Northwestern University, Chicago, IL, USA; ^4^ Department of Pathology, Messejana Heart and Lung Hospital, Fortaleza, Brazil

**Keywords:** adrenocortical carcinoma, targeted therapy, IGF-1R, β-catenin, VEGFR

## Abstract

Adrenocortical carcinoma (ACC) is a rare disease with an estimated incidence of only 0.7 new cases per million per year. Approximately 30-70% of the patients present with advanced disease with very poor prognosis and without effective therapeutic options. In the recent years, unprecedented progresses in cancer biology and genomics have fostered the development of numerous targeted therapies for various malignancies. Immunotherapy has also transformed the treatment landscape of malignancies such as melanoma, among others. However, these advances have not brought meaningful benefits for patients with ACC. Extensive genomic analyses of ACC have revealed numerous signal transduction pathway aberrations (e.g., insulin growth factor receptor and Wnt/β-catenin pathways) that play a central role in pathophysiology. These molecular alterations have been explored as potential therapeutic targets for drug development. This manuscript summarizes recent discoveries in ACC biology, reviews the results of early clinical studies with targeted therapies, and provides the rationale for emerging treatment strategies such as immunotherapy.

## INTRODUCTION

Adrenocortical carcinoma (ACC) is an exceedingly rare malignancy with no effective standard treatment options. ACC is responsible for 0.2% of all cancer deaths in the United States and has an estimated incidence of 0.7 cases per million per year.[1] The disease incidence has a bimodal distribution with peaks in the first and fourth decades of life, and a female to male ratio 1.5-2.5:1.[2, 3] A growing number of patients are noted to have asymptomatic, indolent adrenal incidentalomas after undergoing abdominal imaging; in this patient population, the estimated incidence of ACC is as high as 5%.[4–7] The pathologic diagnosis of ACC is based on multiple morphologic parameters that are suggestive but not pathognomonic of malignancy.[8–10] The most widely used diagnostic score are known as Weiss criteria, with several updates, and includes the following parameters: mitosis, atypical mitosis, necrosis, venous invasion, sinusal invasion, capsular invasion, nuclear atypia, diffuse architecture, and clear cell change.[8, 9] Molecular analyses have been recently attempted in helping diagnosis, in particular hypermethylation, miRNA, *TP53*, *ZNRF3*, β-catenin are possible candidates that could help diagnose and establish prognosis, but are not yet used in current pathologic diagnosis.[11] About 40-60% of patients with malignant tumors of the adrenal glands present with symptoms related to excessive hormonal production such as hypercortisolism or hyperandrogenism.[2, 12]

Most cases of ACC are sporadic with no identifiable risk factor. Rarely, ACC can be part of tumor hereditary syndromes associated with specific germ line mutations, namely Li-Fraumeni (*TP53* gene mutation) or Lynch syndrome (mutations in various mismatch repair genes).[13–15] Case reports have also suggested an increased risk of ACC in multiple endocrine neoplasia syndrome type 1 (MEN1) and familial adenomatous polyposis.[16] Surgery is the treatment of choice for patients presenting with resectable disease and is often followed by adjuvant chemotherapy with mitotane to mitigate the risk of disease recurrence in high-risk patients despite the lack of prospective data to support this approach.[17] The group of approximately 30-70% of patients presenting with unresectable metastatic disease carry an extremely poor prognosis related to the aggressive biological behavior and the lack of effective therapeutic options.[2, 12] Mitotane, which contains cytotoxic and anti-steroid synthesis activity properties, has been the corner stone of the treatment of advanced disease for decades.[18, 19] Limited benefit from polychemotherapy contributes to the dismal prognosis of advanced disease with an estimated 5-year survival rate of less than 15%.[20]

Major advances in the understanding of the genetic pathophysiology of cancer have led to significant advancement in the treatment of several malignancies including the development of effective targeted therapies.[21] Investigation of the genomic landscape of ACC revealed that it is a biologically and genetically heterogeneous malignancy with transcriptome clusters associated with distinct clinical behaviors.[11] These studies also demonstrated potential therapeutic targets that will be reviewed in this manuscript.

## CURRENT TREATMENT APPROACH

Mitotane, the only FDA-approved drug for ACC, displays single-agent activity (10-30% tumor response rates) based on its adrenolytic activity but its broad clinical use is challenged by an unfavorable toxicity profile.[22–24] The published trials on mitotane monotherapy activity are for the most part retrospective. Only two prospective trials have been reported with less than 20 patients each with no clear survival benefit.[25, 26] Polychemotherapy has also limited efficacy in advanced disease with small-uncontrolled studies showing response rates of 10-33%.[27–29]

There have been a few studies of potential biomarkers to help identify patients who may benefit from mitotane and/or platinum-based chemotherapy. In the adjuvant setting, establishing the tumor proliferation index by Ki-67 labeling, with a cutoff of 10% dividing two groups of patients with high and low risk, may help in the decision to use mitotane alone or in combination with other drugs.[30–32] A few studies have also addressed the expression of ribonucleotide reductase large subunit and excision repair cross-complementation group 1 (ERCC1) in ACC at the protein level by immunohistochemistry, and determined the association of the expression with clinical outcome and response to mitotane and cytotoxic therapy, but the results are inconsistent.[33–35]

The only chemotherapy based phase III randomized clinical trial ever conducted in advanced ACC showed that mitotane combined with etoposide, doxorubicin and cisplatin (EDPM) provided additional clinical benefit compared to mitotane plus streptozocin.[36] The patients randomized to receive EDPM had higher tumor response rates when compared to patients treated with streptozocin combined with mitotane (23% *vs*. 9%). Prolongation of progression-free survival (PFS) was also observed in the EDPM arm (5 *vs*. 2 months). There was no significant benefit in overall survival (OS) (14 *vs*. 12 months). It is important to emphasize that up to 58% of patients receiving EDPM had serious adverse events compared to 41% in the mitotane plus streptozocin arm.

The cytotoxic mechanism of action as well as resistance mechanisms to mitotane are not well understood.[24] Mitotane is also generally not well tolerated as monotherapy and levels > 14 mcg/ml are associated with better antitumor response.[37] It is important to review that mitotane is a strong cytochrome P450-3A4 (CYP3A4) inducer and this may lead to significant drug interactions and decrease the activity of combination strategies.

The results of the FIRM-ACT phase III trial showing modest improvement of disease control with polychemotherapy and the high frequency of serious adverse events stresses the pressing need for more effective therapies. Furthermore in light of the rate of serious adverse events there is no clear evidence that a combination of cytotoxic agents with mitotane is better than a sequential strategy.

## POTENTIAL THERAPEUTIC TARGETS

### Insulin growth factor (IGF)/mTOR signaling

Insulin growth factor receptor 1 (IGF-1R) is a transmembrane cellular receptor which has a heterotetrameric structure, characterized by two ‘half receptors’, each of which in turn comprises a predominately extracellular α-chain that is involved in ligand binding and a predominately intracellular β-chain that includes the tyrosine kinase domain.[38]

**Figure 1:**
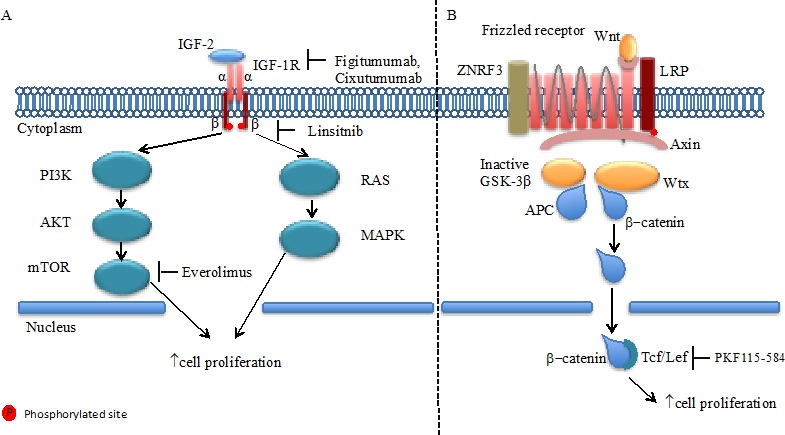
IGF-1R and Wnt/Frizzled receptor pathways **A.** In adrenocortical carcinoma cells IGF2 binds to IGF1 receptor (IGF1R) family. IGF-1R has a tetrameric structure in which the intra-cellular β-chain tyrosine kinase activity regulated by ligand binding to extra-cellular α chain. Downstream of these receptors are the well-known Akt and MAPK intracellular signaling networks, which when activated promote cell proliferation. **B.** The Wnt proteins, by binding to frizzled receptors and the LRP co-receptor, act to suppress the activity of glycogen synthase kinase-3β (GSK-3β). ZNRF3 promotes degradation of Wnt receptor functioning as tumor suppressors. This prevents phosphorylation of downstream molecules allowing β-catenin association with Tcf/Lef in the nucleus and subsequent increased cell proliferation.

Activation of the IGF-1R by one of its ligands insulin-like growth factor 2 (IGF2), encoded by a gene that is located among a gene cluster on chromosome 11p15 and that undergoes genomic imprinting, results in activation of the PI3K/AKT/mTOR cascade and the RAS-MAPK pathway leading to stimulation of protein translation and cell proliferation (Figure 1).[39]

Chromosome 11p15 abnormalities are present in up to 90% of sporadic ACCs and IGF2 is commonly overexpressed in this tumor.[40] In fact both IGF2 mRNA and protein are overexpressed in more than 90% of ACC, with a good correlation between mRNA and protein.[41] Mounting evidence suggest that overproduction of IGF2 is due to uniparental disomy or loss of imprinting of the maternal allele.[42, 43] Moreover, *IGF2* is expressed differently in ACC when compared adrenocortical adenomas or normal adrenal cortex tissue.[44] It has been hypothesized that IGF2 overexpression is low or absent in the beginning of oncogenesis, which may suggest that other signaling pathways may also play a role in the ACC tumorigenesis.[43]

Evidence also suggests that the IGF-1R plays a pivotal role in ACC pathophysiology representing an important therapeutic target in advanced ACC.[43, 45] Indeed, the *IGF2* gene and protein expression has been associated with aggressive clinic-pathological features of ACC when assessed by immunohistochemistry and gene expression arrays (*i.e.,* high grade and metastatic disease).[44, 46] These results provided the rationale and fostered the clinical development of anti-IGF antibodies for treatment of this disease.

Linsitinib, an oral small molecule inhibitor of both the IGF-1R and the insulin receptor, generated significant enthusiasm based on early clinical results. In a phase I trial, 14 patients with refractory ACC were treated with 3 intermittent dosing schedules of linsitinib.[47] Two out of the 9 patients had partial responses and remained in the study for 703 and 199 days, respectively. A double-blind, placebo-controlled phase III clinical trial evaluated the efficacy of linsitinib in 139 previously treated patients with advanced ACC.[48] The study was prematurely stopped due to the failure of linsitinib to improve PFS or OS compared to placebo (median OS: 323 *vs*. 356 days; HR 0.94; *p* = 0.77).

Of note 90% of patients in the linsitinib arm had been exposed to mitotane based chemotherapy in the metastatic setting. A short mitotane wash out period could have affected linsitinib efficacy due interaction through CYP3A4.[49] Also despite reported equivalent levels of IGF-1 in active linsitinib and placebo groups question remains if linsitinib reached its target. Finally, IGF-1R might not be a major driver of adrenocortical carcinoma. Overexpression of IGF-2 could represent a bystander effect caused by dysregulation of another gene at 11p15, including, *p57Kip2* or other transcripts at the loci.[50, 51] Finally, ACC could be driven by multiple pathway aberrations requiring combination targeted therapies as discussed below.

Figitumumab, a fully human IgG2 monoclonal antibody that targets the IGF-1R, was tested in a phase I dose-escalation study in patients with advanced solid tumors.[52, 53] After a total of 50 cycles of figitumumab administered, no responses were documented among the 14 patients with previous treated metastatic ACC and no patients had stable disease lasting more than 6 months. Cixutumumab (IMC-A12), another antibody with high affinity to IGF-1R, was evaluated in combination with mitotane as first-line treatment among 20 patients with metastatic ACC. A partial response was observed in one patient (5%) and the trial was terminated due to slow accrual and low response rates. The most common toxicities mirrored the ones commonly seen with mitotane alone, namely diarrhea and neurological complications.[54] Cixutumumab was also tested in combination with the mTOR inhibitor temsirolimus for the treatment of 42 patients with advanced solid tumors in a phase I trial. Four out of 10 patients with ACC had stable disease lasting longer than 8 months, prompting the opening of an ACC expansion cohort that included 26 patients.[55] There were no partial or complete responses, but 42% achieved stable disease for at least 6 months. The combination was relatively well tolerated with toxicities known to be associated with temsirolimus including mucositis, thrombocytopenia, hypertrygliceridemia, hypercholesterolemia, and hyperglycemia.[56]

These results highlight the therapeutic potential of combinatorial strategies and the possible role of mTOR inhibitors. In fact, De Martino et al. recently evaluated the expression of mTOR and downstream molecules in ACC and showed activation of the pathway in a subset of tumors.[57] Nevertheless, single-agent everolimus, another mTOR inhibitor, showed no signal of clinical activity in an exploratory study of 4 patients with advanced ACC.[58]

### Vascular endothelial growth factor receptor pathway

The family of vascular endothelial growth factor receptors (VEGFRs) plays a central role in tumor angiogenesis. Of the three VEGFRs, VEGFR1 (fms-like tyrosine kinase-1), VEGFR2 (fetal liver kinase-1/kinase insert domain receptor), and VEGFR3 (fms-like tyrosine kinase-4), VEGFR2 has the most significant role in mediating the growth of blood vessels necessary for tumor growth.[59] Activation of the VEGFR pathway has been well documented in ACC and provides the rationale for targeting these receptors.[60, 61]

Axitinib (AG-013736) is an oral, potent, and selective oral inhibitor of VEGFR tyrosine kinases 1, 2, 3.[62] In phase II study, axitinib administered to 13 patients with metastatic or locally advanced ACC resulted in no responses.[63] Sorafenib, an inhibitor of several tyrosine kinase receptors such as VEGFR2, VEGFR3, platelet-derived growth factor receptor (PDGFR), and RAF-1, was investigated in a phase II trial in combination with paclitaxel for patients with advanced disease and showed no clinical activity. [64] [65] Sunitinib, another multikinase inhibitor with activity against VEGFR and PDGFR, was also investigated in a phase II trial with 35 patients with refractory ACC.[66] There were no documented tumor responses and the median PFS was 2.8 months.[67] However sunitinib serum levels might have been reduced by mitotane induced CYP3A4 activity attenuating its antitumor activity and adverse effects.

Lastly, bevacizumab, an anti-VEGF antibody, was given in combination with capecitabine to 10 patients with locally advanced or metastatic disease after treatment with mitotane and at least one line of chemotherapy led to no meaningful clinical benefit was observed.[68] These results document the limited clinical efficacy of these VEGF and VEGFR inhibitors for the treatment of ACC. Nonetheless pre-clinical data suggest increased efficacy of antiangiogenic therapy with sunitinib when combined with ERK pathway inhibitor in ACC indicating that further antiangiogenic therapy may indeed show antitumor efficacy.[69]

### Platelet-derived growth factor receptor (PDGFR) signaling pathway

Platelet-derived growth factor (PDGF) was one of the first identified polypeptide growth factors identified that signals through a cell surface tyrosine kinase receptor to stimulate various cellular functions including growth, proliferation, and differentiation. Since then, several related genes have been identified constituting a family of ligands (primarily PDGF-α and -β) and their cognate receptors (PDGFR-α and -β) have been identified. PDGFR expression has been demonstrated in a number of solid tumors including ACC.[70, 71] These results suggested that imatinib, a small molecule inhibitor of the c-ABL, PDGFR and stem cell ligand receptor (c-KIT) tyrosine kinases, could potentially have activity in ACC. A phase II study testing imatinib in patients with metastatic solid tumors expressing PDGFR or c-KIT included 4 patients with metastatic ACC, but none of those patients showed clinical response to therapy.[71] On the other hand, another study exploring the combination of imatinib with dacarbazine and capecitabine, in patients with advanced endocrine malignancies documented tumor responses in 2 out of 7 patients with metastatic ACC.[72]

### Epidermal growth factor receptor (EGFR) signaling

EGFR family is part of a complex signal-transduction network that is central to several critical cellular processes.[73] EGFR has been show to be overexpressed in ACC when compared to adrenal adenomas and normal tissue suggesting the potential use of EGFR expression as a marker of malignancy.[74, 75]. EGFR amplification by FISH and polysomy of chromosome 7 are noted to be more frequent in ACC specimens compared to adrenocortical adenomas.[76] Sequencing of the *EGFR* gene in 30 cases did not reveal mutations that are usually associated with response to EGFR tyrosine kinase inhibitors such as erlotinib.[75] Nevertheless, inhibition of EGFR signaling was able to reduce cell viability in ACC lines *in vitro*.[77] Ten patients with heavily pretreated metastatic ACC were treated with erlotinib and gemcitabine, but clinical activity was seen in only one patient with reduction in the size of liver metastasis leading to PFS of 8 months. [78] Despite the frequent expression of EGFR, the rarity of activating mutations predictive of response to EGFR tyrosine kinase inhibitors (TKIs) makes unlikely that this class of drugs will be clinically effective for ACC.

### Fibroblast growth factor receptor (FGFR) signaling

Genetic studies have identified disturbances in other signaling pathways such as the FGFR cascade.[79, 80] For instance, FGFR4 has been found to be overexpressed in ACC, particularly in the pediatric population.[81] FGFR4 expression was assessed in 57 ACC tumors with overexpression demonstrated in 65% of specimens. In adults, FGFR4 overexpression and amplification (identified in 13% of pediatric tumors and 30% of adults) was associated with worse outcomes.[82] A phase II trial investigating the efficacy of dovitinib, a tyrosine kinase inhibitor with nonselective activity against the FGFR, included 17 patients with unresectable ACC.[83] One partial response was documented but 23% of patients achieved stable disease lasting >6 months. These preliminary results support the current investigation of the potential role of selective FGFR inhibitors for treatment of ACC, particularly in tumors with FGFR overexpression or carrying other genetic aberrations such as amplifications, activating mutations or gene fusions.

### Inhibitors of steroidogenesis

Acetyl-CoA acetyltransferase 1 (ACAT1) catalyzes cholesterol ester formation from cholesterol and long-chain fatty acyl-CoA in adrenal glands and is important in creating a reservoir of substrate for steroid biosynthesis making this enzyme a potential therapeutic target for ACC. A selective inhibitor of ACAT1 (ATR-101-001) is currently undergoing investigation in a phase I study recruiting patients with advanced ACC (NCT01898715).[84, 85] Based on the premise that many ACCs require substantial intracellular cholesterol as a substrate for steroidogenesis, drugs capable of disrupting cholesterol uptake have therapeutic potential in ACC. In fact, synthetic high-density lipoprotein (HDL) nanoparticles that inhibit the cholesterol transporters SR-BI inhibited cortisol production in ACC cell lines and enhanced the apoptosis induced by etoposide, cisplatin or mitotane.[86] Synthetic HDL nanoparticles are undergoing active investigation as novel anti-cancer agents and hold promise for the treatment of ACC.[87, 88]

Steroidogenic factor 1 (SF-1) is a nuclear transcription factor critical in the development of steroidogenic tissues such as the adrenal glands.[89] SF-1 also induces proliferation of ACC cell lines and tumor growth *in vivo*.[90] SF-1 inhibitors inhibited the proliferation elicited by SF-1 overexpression *in vitro* suggesting a possible therapeutic potential.[91] Furthermore, increased expression of SF-1 among 167 ACC tumors was associated with worse prognosis independent of tumor staging.[92] The ongoing expansion of knowledge regarding SF-1 regulated genes may lead to novel therapeutic targets.[93]

## FUTURE POTENTIAL TARGETS

### Wnt/β-catenin

β-catenin is an important component of the adrenal cortex differentiation through the Wnt signaling pathway.[94] After translocation into the nucleus, β-catenin along with the T cell factor/lymphoid enhancing factor (Tcf/Lef) family of transcription factors induces Wnt target gene expression.[95, 96] The most common genetic alteration identified among 77 ACC tumors by exome sequencing and single nucleotide polymorphism (SNP) array analysis were homozygous deletions in the *ZNRF3* gene.[11] This gene encodes a cell surface E3 ubiquitin ligase and is a potential new tumor suppressor gene related to the β-catenin pathway. Other mutations in genes of the β-catenin pathway detected in ACC included *APC* (2%) and *CTNNB1* (β-catenin gene) (16%) found in 30% of analyzed tumors.[11] In addition, β-catenin nuclear expression assessed by immunohistochemistry and the presence of mutations in the *CTNNB1* and *APC* genes represented independent prognostic factors in patients with resected ACC.[97] The therapeutic potential of this pathway was suggested by pre-clinical data showing that a small-molecule inhibitor of the Tcf/β-catenin complex (PKF115-584) induced apoptosis in ACC cell lines carrying mutations in β-catenin.[98] While there have been significant advances in therapeutic strategies targeting the Wnt pathway, we are not aware of any preliminary clinical results or ongoing trials specific for ACC.[99] Conversely, pre-clinical data support that Wnt/β-catenin pathway targeted therapy with anti-Wnt monoclonal antibody can induce apoptosis in a wide variety of cancer cells and suppress tumor growth in xenograft model.[100, 101] In addition, interactions between the Wnt/β-catenin and other important intracellular pathways have been proposed. For instance, inhibition of γ-secretase, which is a key component in the Notch extra-cellular signal transduction pathway showed significant anti-tumor activity in patients desmoid tumors (ORR 71.4%) in a phase I trial.[102, 103] *CTNNB1* mutations are observed is as much as 85% of desmoid tumors, which may indicate potential interactions between the Notch and Wnt/β-catenin pathways.[104]

### DNA methylation

DNA methylation has been studied in ACC.[40] The methylation patterns of CpG islands in promoter regions were analyzed in 51 ACC and 84 adrenal adenoma specimens. Two unsupervised hierarchical clusters of methylation profiles were identified and the global level of methylation was significantly associated with survival. Among the 25 genes with an inverse correlation between methylation and expression were *H19, GSTM1, GSTP1, G0S2*.[105] These findings are consistent with results showing reduced expression of *H19* in ACC as a function of loss of maternal allele with the duplication of paternal allele, leading to biallelic expression of IGF2 gene and decrease in *H19* and p57kip2 expression.[45, 50] Also, ACC cells lines were treated with the cytosine methylation inhibitor 5-aza-2′-deoxycytine as an attempt to reduce the methylation of *H19* promoter and thereby increase its expression. Indeed, the treatment increased H19 mRNA expression, inhibited IGF-2 mRNA and decreased cellular proliferation.[106] These results provide a rationale to further investigate the efficacy of DNA demethylating agents in ACC.

### Estrogen receptors

There is evidence in solid tumors of an interaction between the IGF signaling pathway and estrogens.[107, 108] ACC cell model (H295R) showed that estrogen receptor ERβ is significantly more expressed then ERα.[109] Tumor tissue PCR and immunohistochemistry based analysis showed that ERβ is present in normal adrenal gland tissue and ACC.[110, 111] In addition, the apoptotic effects of 4-hydroxytamoxifen in H295R cells may be consequent to the enhanced levels of ERβ which stimulate the expression of FasL interacting with activating protein (AP)-1 sites located within its promoter sequence.[109] Further exploration of the ER pathway and its interaction with other cellular biologic processes in ACC may be of potential interest.

### Hepatocyte growth factor (HGF)/c-Met pathway

c-Met is a transmembrane tyrosine kinase receptor naturally activated by its ligand HGF. Activation of this pathway through mutations or overexpression can lead to cellular proliferation and metastasis in several solid tumors.[112] Also, c-Met expression in solid tumors has been associated with a more aggressive cancer phenotype.[113–115] Transcriptome analysis of ACC cells documented activation of the c-Met pathway in ACC cells, and treatment of ACC cells with HGF increased cell proliferation both *in vivo* and *in vitro*.[116] Consistent with these results, cabozantinib, a tyrosine kinase inhibitor with activity against VEGF-R and c-Met inhibited ACC tumor growth *in vivo*.[116] To the best of our knowledge, there are no clinical data showing efficacy of c-Met pathway inhibitors for the treatment of ACC, but these preliminary results support further investigation.

### MicroRNAs

MicroRNAs (miRNAs) are noncoding RNA molecules that are involved in cellular processes related to post-transcriptional regulation, and some have recently been correlated with the development and progression of various neoplasms.[117] In ACC, global alterations and deregulations in the expression of miRNAS have been reported.[118, 119] While most studies have attempted to identify miRNAs that could help to distinguishing adenomas from ACC or other tumors, a few recent studies have addressed the potential roles of these molecules in predicting response to therapy. For example, both miR-483-5p and miR-7, have been shown to regulate the expression of apoptotic pathways, making these molecules attractive for anticancer therapy.[120, 121]

### Immune system

Studies investigating immunotherapy strategies in ACC provide preliminary evidence that anti-tumor immune response could be of relevance. Based on the wide expression of the steroidogenic acute regulatory protein (StAR) in adrenal tissue and ACC cell models, a specific immune response against this antigen was elicited using an immunization protocol with DNA plasmids and recombinant vaccinia virus vectors and resulted in an anti-tumor effect in a xenograft model utilizing tumor cell line expressing StAR.[122–125] Another study describing vaccination of two patients with metastatic ACC using autologous dendritic cells pulsed with autologous tumor lysate showed tumor-specific immune response in spite of excessive production of glucocorticoids by the tumor.[126] The anti-neoplastic activity resulting from immunotherapy strategies (*e.g.,* checkpoint inhibitors anti-CTLA-4 and anti-PD-1 antibodies) for the treatment of solid tumors such as melanoma and non-small cell lung cancer has driven the interest to explore its potential clinical efficacy in ACC.[127–130] Tumor membrane and tumor infiltrating mononuclear cells were assessed for PD-L1 expression through immunohistochemistry in 27 patients with resected ACC. There was no correlation between PD-L1 expression and overall survival, higher tumor grade or stage at diagnosis.[131] The role of immune checkpoints in the pathophysiology of ACC and its possible therapeutic implication remains to be determined. Clinical trials evaluating this strategy will be soon recruiting patients (NCT02673333, NCT02720484 and NCT02720484).[132–134]

Another potential target is interleukin-13 receptor α2 (IL-13Rα2), which is a high-affinity receptor for the Th2-derived cytokine interleukin-13 (IL- 13).[135] IL-13Rα2 is significantly overexpressed in ACCs as compared to normal adrenocortical tissues and benign adrenocortical tumors.[136] IL-13-PE is a chimeric fusion protein consisting of human IL-13 and a truncated form of Pseudomonas exotoxin A.[136] A phase I trial of IL-13-PE in ACC expressing IL-13Rα2 showed stable disease among 3/6 patients lasting for 2-5.5 months[137] IL-13-PE at dose of 1-2 lg/kg was administered intravenously (IV) on day 1, 3, and 5 in a 4-week cycle. Dose-limiting toxicities were observed at 2 lg/kg, at which patients exhibited thrombocytopenia and renal insufficiency without requiring dialysis. A possible limitation is that neutralizing antibodies developed in 67% of the patients in high titers.

## DISCUSSION

As of today, chemotherapy remains the standard of care treatment for the treatment of locally advanced or metastatic ACC. The FIRM-ACT indicated that combination of mitotane with etoposide, doxorubicin and cisplatin (EDPM) promotes PFS prolongation in patients with metastatic ACC compared to treatment with mitotane plus streptozocin.[36] Nonetheless, EDPM is associated with serious adverse events in greater than 50% of patients including myelosuppression, cardiovascular or thromboembolic events, infection, neurotoxicity and general health deterioration. For patients with good performance status, EPDM seems to be a reasonable option, whereas mitotane combined with streptozocin could be offered to patients with poorer performance status.[17]

**Figure 2:**
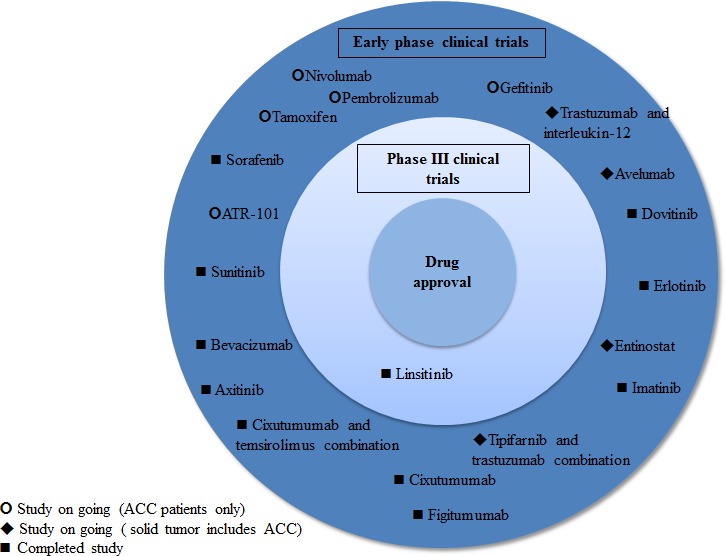
Targeted agents and immune checkpoint inhibitors studied and under development in ACC

As reviewed above, many early phase studies have been conducted in ACC in the past years, but no breakthrough targeted therapy trial has been reported thus far (Figure 2). IGFR, while a promising target given the high IGFR/mTOR pathway activation in ACC, failed as predictive marker of benefit with anti IGFR therapy.[40] The phase III trial of linsitinib *versus* placebo in ACC was closed due to the lack of demonstration of overall survival benefit at interim analysis.[48] The simultaneous targeting of the IGFR and mTOR showed more promising results in one phase I trial with 11 out of 26 patients experiencing stable disease after 6 months of therapy, suggesting potential efficacy from combinatory strategies.[55] Agents targeting the VEGFRs and other tyrosine kinase receptors such as cKIT, EGFR and FGFR have not shown significant antitumor activity. [63, 65, 67, 68, 72, 78, 83] While interesting pre-clinical data of other potential targets for ACC (*e.g.,* steroidogenic factor 1, c-Met pathway, estrogen receptors, DNA methylation) are emerging, an expansion of immunotherapy translational studies would also be desirable.[92, 106, 109, 116, 131]

Importantly, several studies have explored the genomic landscape of this rare malignancy using next generation sequencing approaches.[11, 79, 138–145] These studies highlight not only the significant tumor heterogeneity inherent to ACC, but they were also able to define groups of different prognosis based on the mutational profiles.[11, 140] In the study by Ross et al, clinical specimens from 29 patients with metastatic or locally advanced adrenocortical carcinoma were sequenced.[138] In 17 (59%) of ACC, at least one genomic alteration was associated with an available therapeutic or a mechanism-based clinical trial. Poli et al reported a different proteomic profile in adrenocortical carcinoma compared with normal adrenal cortex characterized by overexpression of mainly metabolic enzymes that can be a potential target for novel therapeutics.[146]

These studies have identified recurring mutations in several genes such as *ZNRF3* (20%), *CTNNB1* (14%), *TP53* (14%), *CDKN2A* (11%), *RB1* (5%), *MEN* (16%), *DAXX* (5%), *MED12* (3%) and *TERT* (5%).[11] Despite the identification of common genetic abnormalities, the development of targeted therapies for this disease has been thwarted by the rarity of ACC as illustrated by the small sample sizes ranging from 8 to 132 tumor samples in these studies characterizing the genomic alterations. In addition, the construction of valid prognostic and predictive multivariable models incorporating clinicopathological and genomic characteristics are also hampered by the scarcity of the ACC tumor tissue. Comprehensive genomic profiling of ACC tumor samples is underway through The Cancer Genome Atlas program (TCGA) and final results are expected in the near future that will certainly add important information to guide future drug development.[147]

Another corollary to the low incidence of ACC is the challenge of conducting prospective biomarker-based clinical trials. As in some instances, ACC represents an underrepresented population of phase I or phase II basket trials (Table 1). Conversely, collaborations have been fostered already such as the European Network for the Study of Adrenal tumors (ENSAT-CANCER) which offers a virtual research environment with a digitally interconnected infrastructure for distributed clinicians specializing in rare adrenal tumors to communicate and collaborate with distributed biomedical research communities.[148] Indeed large studies such as the FIRM ACT trial epitomize the successful endeavor of ACC treatment development insofar it enrolled 304 patients with ACC from 12 different countries.

**Table 1:** Current ongoing studies on advanced ACC at www.clinicaltrials.gov^*^

Drug	Target	Phase	*n*	Study population	Clinicaltrials.gov ID
ATR-101	Acyl-coenzymeA:cholesterol O-acyltransferase	I	59	ACC	NCT01898715
Gossypol acetic acid	Bcl-2	II	29	ACC	NCT00848016
TKM-080301	Polo-like Kinase	I/II	68	Solid tumor allows ACC	NCT01262235
Avelumab	Programmed cell death-ligand 1	I	Not provided	Solid tumor allows ACC	NCT01772004
Gefitinib	Epidermal growth factor receptor	II	33	ACC	NCT00215202
Tamoxifen and cisplatin/doxorubicin	Estrogen receptor	II	30	Solid tumor allows ACC	NCT00002608
Interleukin-12 and trastuzumab	Immunomodulation and Human epidermal growth factor receptor 2 (Her2)	II	15	Solid tumor allows ACC	NCT00004074
Tipifarnib and trastuzumab	Farnesyltransferase	I	24	Solid tumor allows ACC	NCT00005842
Entinostat	Deacetylase	I	75	Solid tumor allows ACC	NCT00020579
ADH-1	N-Cadherin	II	Not provided	Solid tumor N-cadherin + allows ACC	NCT00264433
Pembrolizumab	Programmed cell death 1 inhibitor	II	39	ACC	NCT02673333

* Accessed at www.clinicaltrials.gov on February 11^th^, 2016

Other international collaborations exist in order to increase the clinicopathological datasets, tissue banking, that ultimately will enable biomarker-based clinical studies. For example, the German Adrenocortical Carcinoma Registry is among those projects in which data will be collected for future consideration of prospective studies and facilitate accrual of patients (NCT00453674).[149] Similarly, the National Cancer Institute (NCI) has initiated both prospective clinical data collection, as well as tissue sample and blood sample acquisition of patients with ACC to further develop the understanding of this malignancy (NCT02015026).[150] Furthermore combination of already available and new targeted therapies is likely to optimize tumor response to treatments in development and ultimately prolong survival in patients with ACC.[151]
